# Supplementary material for: A pan-cancer genome-wide analysis reveals tumour dependencies by induction of nonsense-mediated decay
Source: Nat Commun. 2017 Jun 26;8:15943. doi: 10.1038/ncomms15943 (PMC5490262; doi:10.1038/ncomms15943)
Supplement: Supplementary Information [file ncomms15943-s1.pdf]

Type of file: PDF
Size of file: 0 KB
Title of file for HTML: Supplementary Information
Description: Supplementary Figures

Type of file: XLSX
Size of file: 0 KB
Title of file for HTML: Supplementary Data 1
Description: A classification of mutations that could result in NMD into NMDelicit and NMD-escape mutations.

Type of file: XLSX
Size of file: 0 KB
Title of file for HTML: Supplementary Data 2
Description: Results of analysis of enrichment of NMD-elicit mutations in hypermutated cancers when compared to other types of mutations.

Type of file: XLSX
Size of file: 0 KB
Title of file for HTML: Supplementary Data 3
Description: Pathway enrichment analysis for genes with NMD-elicit mutations in hypermutated cancers.

Supplementary Information

Supplementary figures

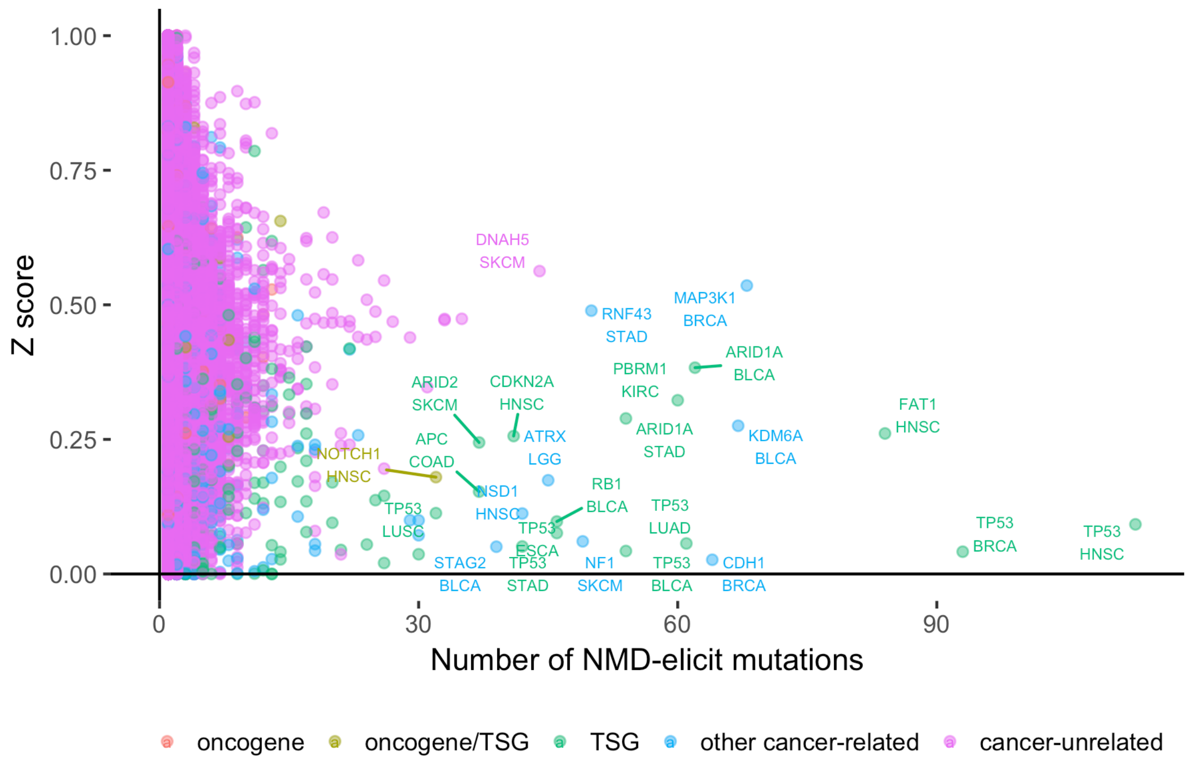

Supplementary Figure 1. Relationship between the frequency and the MARE of NMD-elicit mutations. A scatter plot showing the frequency (x axis) and the magnitude of NMD-associated reduction in expression (z scores) of NMD-elicit mutations targeting individual genes across all cancer types. Note that a z score $\geqslant$ 0.5 indicates no reduction in expression and that a low z score indicates larger magnitude of NMD associated reduction in expression.

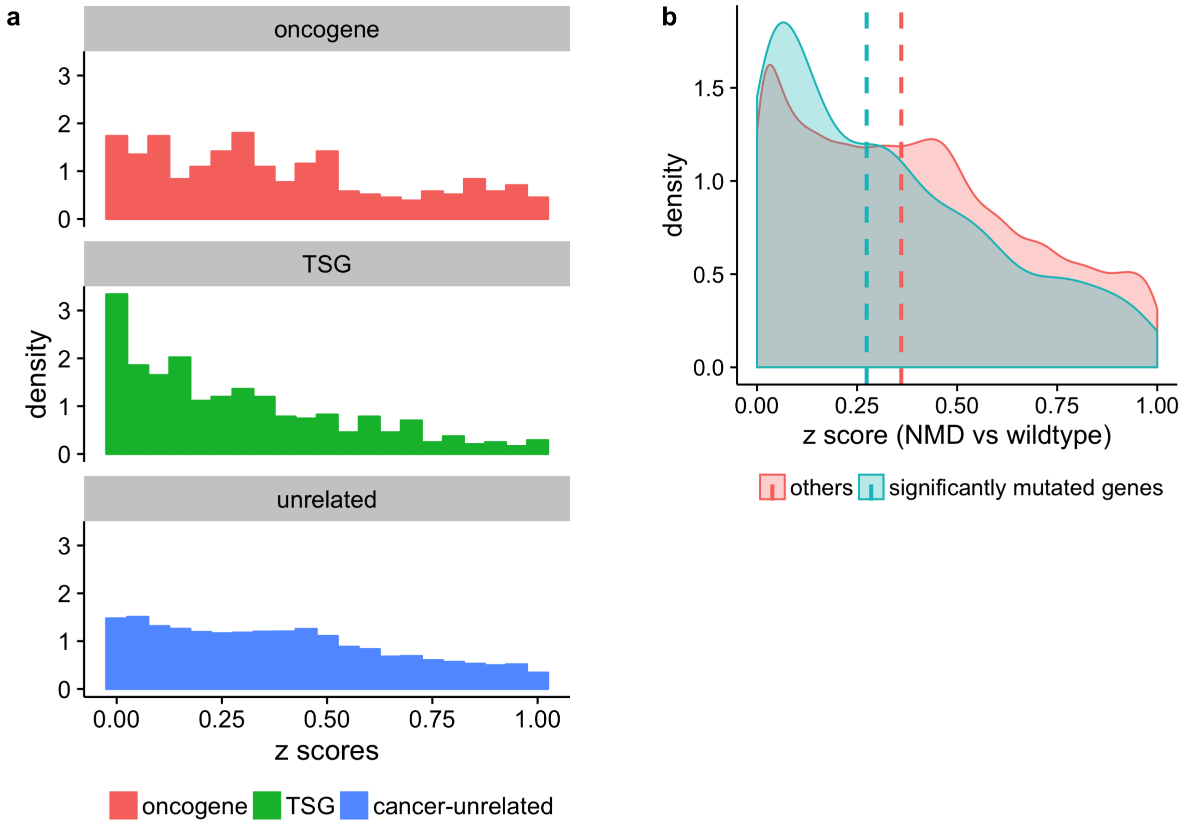

Supplementary Figure 2. Tumour suppressor genes are frequently targeted affected by NMD in cancer. (a) Tumour suppressor genes (TSG) have lower z scores than the genes unannotated in the COSMIC database (ratio = 0.29/0.39, P < 2.2e-16, one-sided t-test), while oncogenes do not (ratio = 0.39/0.39, P = 0.4, one-side t-test). (b) The distribution of z scores of cancer-related significantly mutated genes (SMGs), compared to the other genes. The dashed lines showed the medians of two groups. SMGs have significantly lower z scores than other genes (ratio = 0.32/0.39, P = 2.5e-11, one-side t-test).

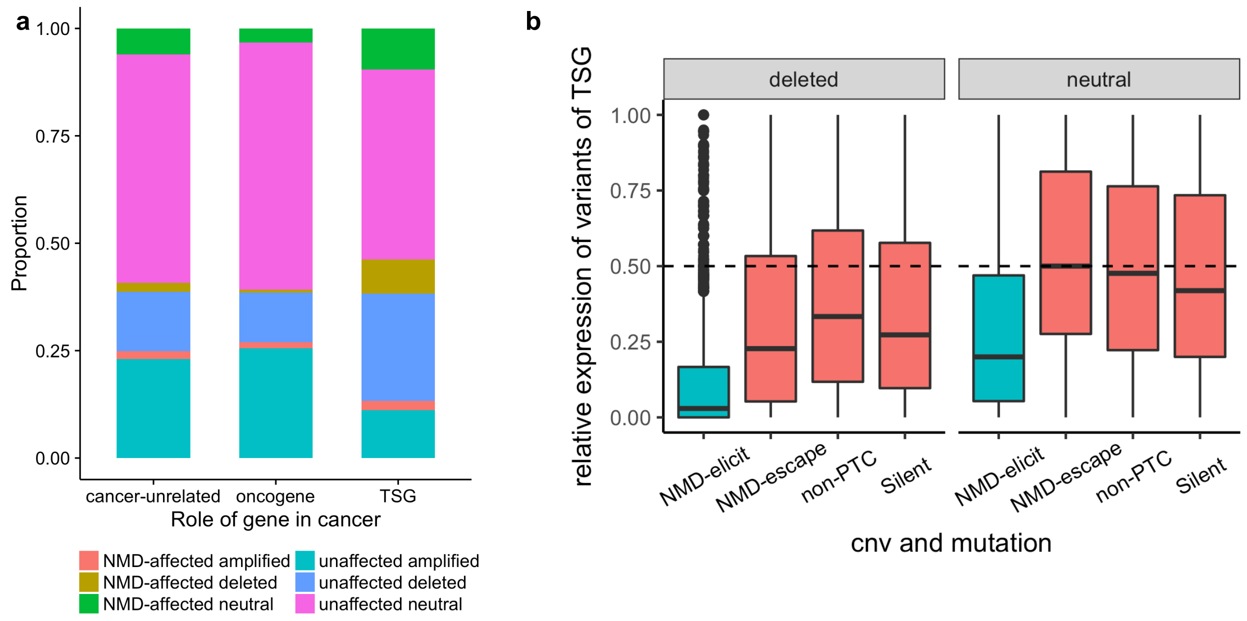

Supplementary Figure 3. NMD-elicit mutations and deletion in TSGs. (a) The co-occurrence of the deletion and NMD-elicit mutations is more frequent in TSGs. A histogram presentation of the proportion of samples having the indicated categories of mutations and CNAs. Unaffected means that the gene in the sample does not harbour NMD-elicit mutations. (b) Relationship between NMD-elicit mutations and expression levels of TSGs in two conditions of copy number variant (CNV, deleted or neutral). NMD-elicit mutations are associated with a deletion of an allele, gene expression is significantly lower compared to the expression of genes that harbour other types of mutations ($P < 2.2e-16$, Mann-Whitney (MWW) test, fold change = 0.09). In the absence of a deletion, NMD-elicit mutations are still associated with a significant reduction of gene expression but at a lower magnitude ($P < 2.2e-16$ by MWW test and fold change = 0.43).

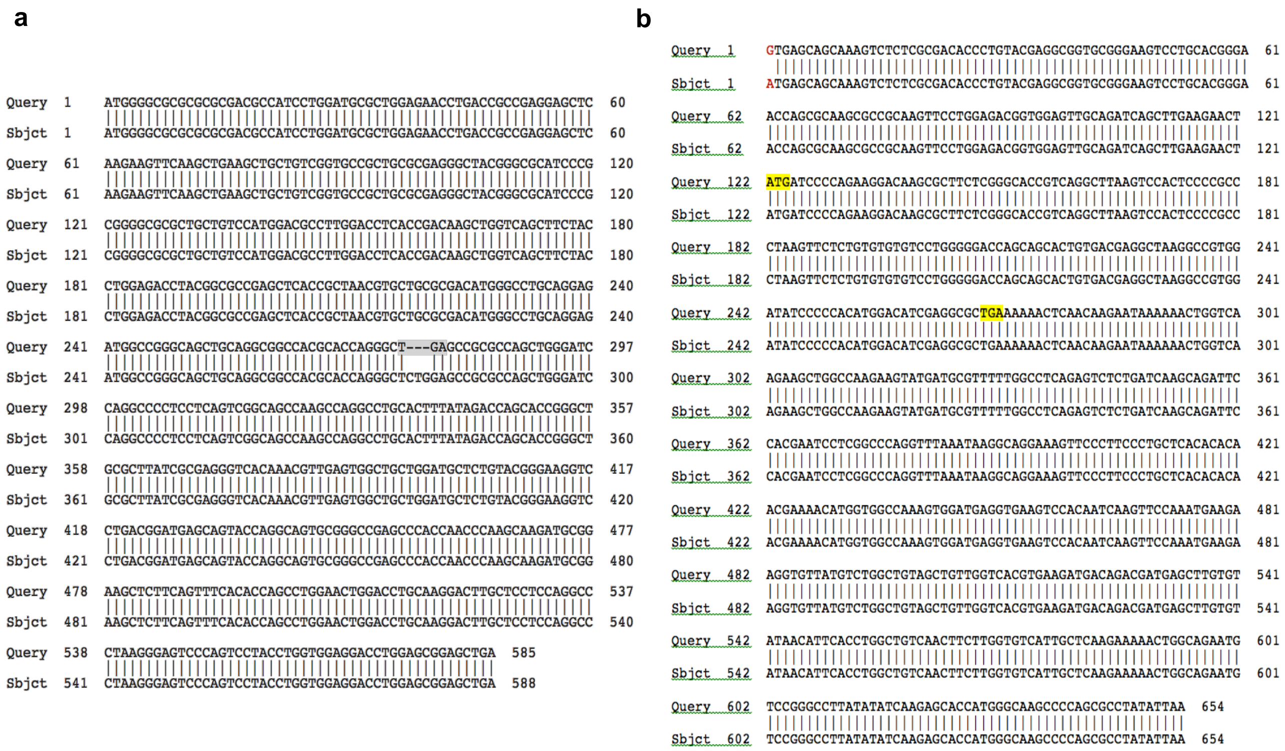

Supplementary Figure 4. Examples of NMD-elicit inframe mutations and missense mutations. two examples explain why some inframe mutations and missense mutations can generate premature stop codons and elicit NMD. The Query 1 is mutated coding sequence and the Sbjct 1 is unmutated. (a) NMD-elicit inframe deletion: this example is a deletion of CTG in sample TCGA-BP-4782-01 at chr16:31213519-31213521 (*PYCARD*). The deletion results in the combination of adjacent T and GA, which is a premature stop codon at 277; this gene has 3 exons and the position of last exon-exon junction is 328. (b) NMD-elicit missense mutations: the example is an A to G mutation in sample TCGA-25-2400-01 at Chr6: 35436212-35436212 in gene *RPL10A*. The mutation changes the original start codon from ATG to GTG. The next start codon is in position 122 and is in a different frame from the original one. This results in a premature stop codon at 269. This gene has 6 exons and the last exon-exon junction is at 483.This is an example of how a missense mutation in the start codon results in a frame shift that is potentially capable of eliciting NMD.
